# Shikonin Inhibits the Proliferation of Human Breast Cancer Cells by Reducing Tumor-Derived Exosomes

**DOI:** 10.3390/molecules21060777

**Published:** 2016-06-16

**Authors:** Yao Wei, Mingzhen Li, Shufang Cui, Dong Wang, Chen-Yu Zhang, Ke Zen, Limin Li

**Affiliations:** State Key Laboratory of Pharmaceutical Biotechnology, Jiangsu Engineering Research Center for MicroRNA Biology and Biotechnology, Nanjing Advanced Institute for Life Sciences, School of Life Sciences, Nanjing University, Nanjing 210093, Jiangsu, China; nju_weiyao@163.com (Y.W.); 15261891180@163.com (M.L.); shufangnice@163.com (S.C.); biowonder@126.com (D.W.)

**Keywords:** breast cancer, proliferation, shikonin, exosome, miR-128, Bax

## Abstract

Shikonin is a naphthoquinone isolated from the traditional Chinese medicine *Lithospermum*. It has been used in the treatment of various tumors. However, the effects of shikonin on such diseases have not been fully elucidated. In the present study, we detected the exosome release of a breast cancer cell line (MCF-7) with shikonin treatment and found a positive relationship between the level of secreted exosomes and cell proliferation. We next analyzed miRNA profiles in MCF-7 cells and exosomes and found that some miRNAs are specifically sorted and abundant in exosomes. Knockdown of the most abundant miRNAs in exosomes and the MCF-7 proliferation assay showed that miR-128 in exosomes negatively regulates the level of Bax in MCF-7 recipient cells and inhibits cell proliferation. These results show that shikonin inhibits the proliferation of MCF-7 cells through reducing tumor-derived exosomal miR-128. The current study suggests that shikonin suppresses MCF-7 growth by the inhibition of exosome release.

## 1. Introduction

Breast cancer is the malignant tumor with the highest incidence among all female malignant tumors [1]. The aetiology of breast cancer is associated with sex-hormone levels, genetic factors and lifestyles [2,3,4,5]. Limitations for breast cancer therapy have created the need to investigate new approaches for treatment [6]. Traditional Chinese medicine has been giving new insights into the therapeutics of cancer and other diseases by providing sources of active compounds, such as artemisinin, a drug against malaria discovered by Dr. Tu Youyou [7].

Shikonin is a natural product isolated from the roots of the Chinese herbs *Lithospermum erythrorhizon*, *Arnebia euchroma* and *Onosma* *paniculata* [8,9,10,11]. The therapeutic effects of shikonin range from anti-inflammatory, anti-oxidant, anti-cancer, wound healing and anti-microbial [12,13,14]. Shikonin can kill cancer cells through a number of mechanisms, including the inhibition of protein tyrosine kinase (PTK) [15]; inhibiting the activities of DNA topoisomerases, which plays a crucial role in cancer cell DNA regulation [16]; and suppression of tumor necrosis factor receptor-associated protein 1 (TRAP1) expression [17]. Other mechanisms of shikonin-induced cancer cell death include increased expression of p53 and inhibition of cancer cell glycolysis via targeting pyruvate kinase M2 (PKM2) [18]. In previous studies, shikonin was shown to inhibit the migration and invasion of breast cancer cells [19]. However, the exact mechanism by which shikonin inhibits breast carcinoma migration and invasion remains unknown.

Exosomal secretion is one of the mechanisms through which tumor cells can communicate with and reprogram their microenvironment [20]. Exosomes are 50–100 nm diameter vesicles formed by the endocytic components of the cells, and they can be secreted by most cells to the extracellular environment [21,22,23]. They mediate the secretion of a wide variety of proteins, lipids, and mRNAs, including microRNAs (miRNAs), and thus, they transmit signals, proteins, lipids, and nucleic acids from cell to cell [24]. Recent research has shown that exosomal miRNAs play a major role in tumor initiation, progression and invasion [25,26,27]. In the present study, we aimed to investigate the effects of shikonin on exosome secretion and the effects of tumor-derived exosomes on tumor cell proliferation. We also aimed to investigate which miRNAs are involved in exosome-mediated proliferation inhibition.

## 2. Results

### 2.1. Shikonin Inhibits the Proliferation of MCF-7 Cells in Time- and Dose-Dependent Manners

**Figure 1:**
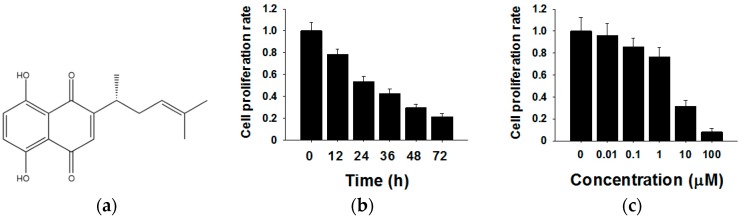
Effects of shikonin on the proliferation of MCF-7 cells. (**a**) The chemical structure of shikonin; (**b**) Shikonin decreases cell proliferation in a time-dependent manner; (**c**) Shikonin decreases cell proliferation in a dose-dependent manner. Data are given as a mean ± SD of individual experiments with three plates for each experiment.

The chemical structure of shikonin is shown in Figure 1a. To investigate the effects of shikonin on human breast cancer cell proliferation, we treated MCF-7 cells for different times (0 h, 12 h, 24 h, 36 h, 48 h and 72 h) or with different concentrations of shikonin (0 µM, 0.01 µM, 0.1 µM, 1 µM, 10 µM and 100 µM). The cell proliferation rate was determined by the CCK8 method. As shown in Figure 1b, the cell proliferation rate decreased 12 h after the 5 µM shikonin treatment, and the inhibitory effects showed time-dependent patterns compared with the 0 h group. Subsequently, MCF-7 cells were exposed to various concentrations of shikonin from 0–100 µM. From the results of CCK8, we found that increased shikonin concentrations improved inhibitory effects on cell proliferation (Figure 1c). These results indicated that shikonin inhibited the proliferation of MCF-7 cells in time- and dose-dependent manners.

### 2.2. Shikonin Inhibits Exosome Release in MCF-7 Cells

**Figure 2:**
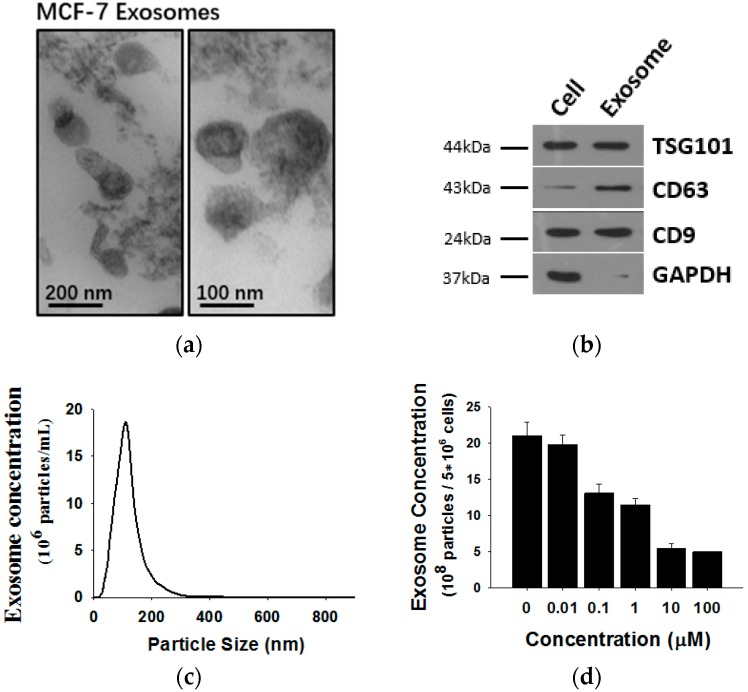
(**a**) Analysis of exosomes released by MCF-7 cells by transmission electron microscopy (TEM); (**b**) western blotting and; (**c**) Nanoparticle Tracking Analysis (NTA); (**d**) It should be noted that exosomes are approximately 100 nm vesicles with a double membrane structure and express marker membrane proteins such as Tsg101, CD63 and CD9. Shikonin decreases exosome release in a dose-dependent manner.

Exosomes released by MCF-7 cells were isolated from cell culture medium and analyzed by transmission electron microscopy (TEM) and Western blotting using antibodies against exosomal marker proteins. As shown in Figure 2, MCF-7 cells release exosomes, double membrane vesicles 50 to 100 nm size, into the culture medium (Figure 2a). The exosomes expressed marker proteins such as CD63, Tsg101 and CD9 but lacked GAPDH (Figure 2b). To monitor the concentration of exosomes released by MCF-7 cells, a NanoSight NS 300 system (NanoSight) was used (Figure 2c). Previous studies revealed that tumor-secreted exosomes are involved in remodeling tumor-stromal interactions and promoting malignancy. Thus, we wondered whether exosome secretion is affected in shikonin-mediated MCF-7 proliferation inhibition. We treated MCF-7 cells with different concentrations of shikonin and detected MCF-7 exosome release. Nanoparticle tracking analysis (NTA) results showed that exosome secretion by MCF-7 cells was decreased after shikonin treatments in a dose-dependent manner (Figure 2d).

### 2.3. Shikonin Inhibits MCF-7 Cell Proliferation by Suppressing Its Exosome Release

**Figure 3:**
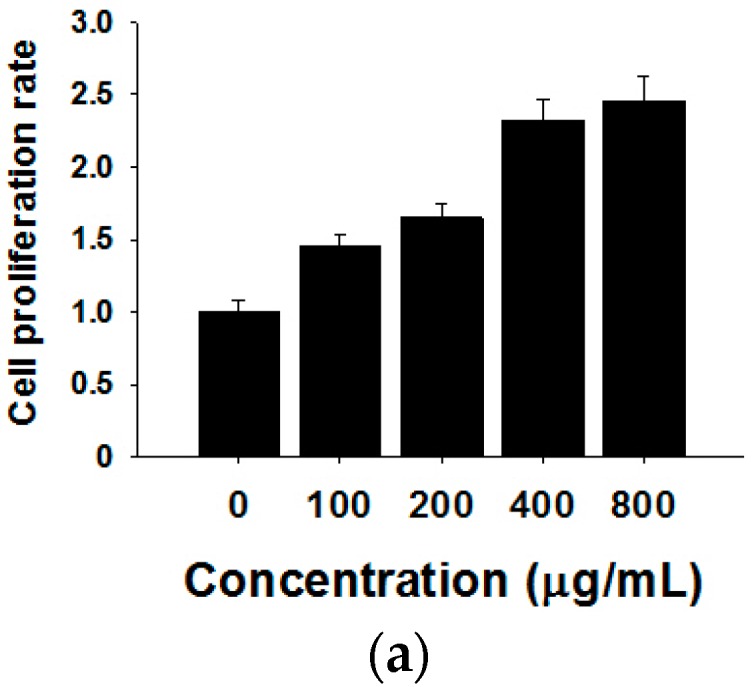

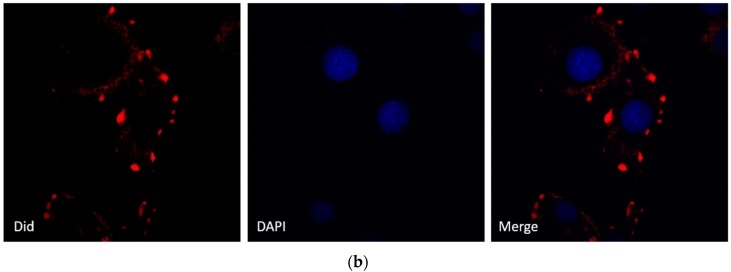
Effects of MCF-7-derived exosomes on proliferation. (**a**) Different concentrations of exosomes from 0–800 µg/mL promote cell proliferation in a dose-dependent manner. The data are collected from three-independent experiments; (**b**) Confocal microscopy visualization of an exosome infused MCF-7 cell line. Secreted exosomes were stained with the cell membrane dye Did. The nuclei were stained with the nuclear dye (DAPI). Merged picture was shown.

To visualize the actual internalization of the exosome transfer from donor into recipient cells, MCF-7 donor cells were stained with the cell membrane dye Did, making possible exosome labelling and in turn visualization. MCF-7 recipient cells and Did labelled exosomes were then incubated for 24 h at 37 °C before evaluation by confocal microscopy. The internalization of exosomes was indicated by a red fluorescent punctuated signal inside the cytoplasm of MCF-7 recipient cells (Figure 3b). To verify the effects of MCF-7 cell-derived exosomes on proliferation, we collected different concentrations of exosomes from donor MCF-7 cells and added them into recipient MCF-7 cells. As shown in Figure 3a, MCF-7-derived exosomes promote recipient MCF-7 cell proliferation in a dose-dependent manner.

### 2.4. Shikonin Decreases Exosomal miR-128 to Inhibit MCF-7 Cell Proliferation

**Figure 4:**
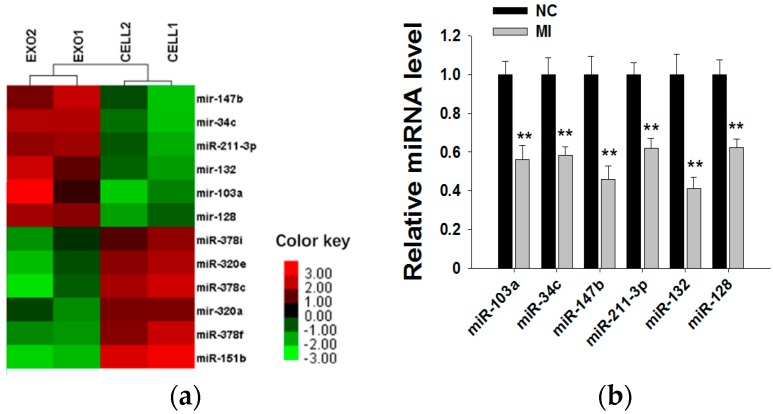

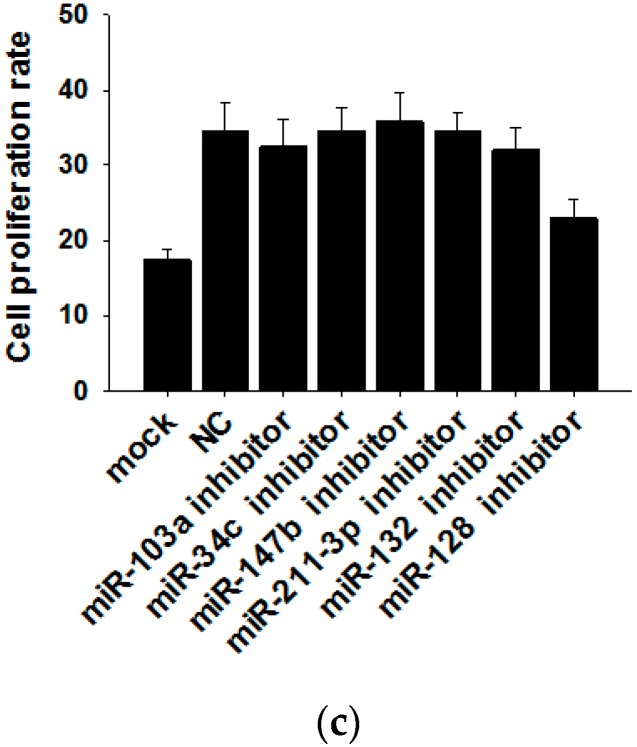
Exosomal miR-128 promotes MCF-7 cell proliferation. (**a**) Microarray analysis of exosomal and cellular miRNAs from MCF-7 cells. The panel shows the heat map of the per-row normalized expression levels of secreted miRNAs differentially expressed in cells *versus* exosomes; (**b**) MiRNA inhibitors can effectively knockdown the levels of six miRNAs in exosomes (the MI group is the miRNA inhibitors of miR-103a, miR-34c, miR-147b, miR-211-3p, miR-132 and miR-128; the NC group is transfected with NC inhibitor, which has the same amount as the basic group but a different sequence from those miRNAs); (**c**) Effects of exosomal miR-128 on MCF-7 cell proliferation (mock group is with no treatment; NC group is transfected with NC inhibitor. ** *p* < 0.01.

Because exosomal miRNAs play a major role in tumor initiation, progression and invasion, we subsequently explored whether MCF-7 cell-derived exosomal miRNAs affect MCF-7 cell proliferation. By searching miRNA microarray assay results of MCF-7 cells and exosomes from Gene Expression Omnibus (accession number: GSE60716), we found that miRNA repertoires of exosomes differ from those of their parental cells, indicating that miRNAs are specifically sorted into exosomes. In Figure 4a, we list the top six miRNAs more highly representing in exosomes than in cells (such as miR-103a, miR-34c, miR-147b, miR-211-3p, miR-132 and miR-128) and the top six miRNAs more highly representing in cells than in exosomes (such as miR-151b, miR-378c, miR-378f, miR-320e, miR-320a and miR-378i). Next, we used six miRNA inhibitors to knockdown the six miRNAs in MCF-7-derived exosomes. After transfection with miRNA inhibitors for 24 h, the expressions of the six miRNAs in exosomes were measured by qRT-PCR (Figure 4b). miRNA knockdown exosomes were individually added into recipient MCF-7 cells, and determination of the cell viability rate was performed by the CCK8 method. As shown in Figure 4c, only miR-128 knockdown exosomes showed decreased effects towards promoting cell proliferation, indicating that exosomal miR-128 has a positive effect on MCF-7 cell proliferation.

### 2.5. miR-128 Promotes MCF-7 Cell Proliferation by Targeting the Bax Gene

**Figure 5:**
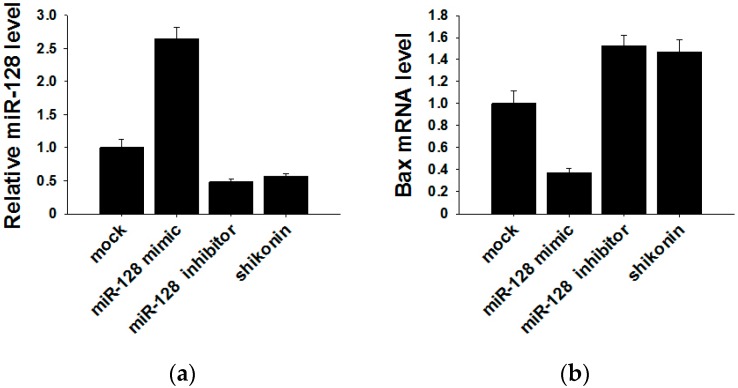

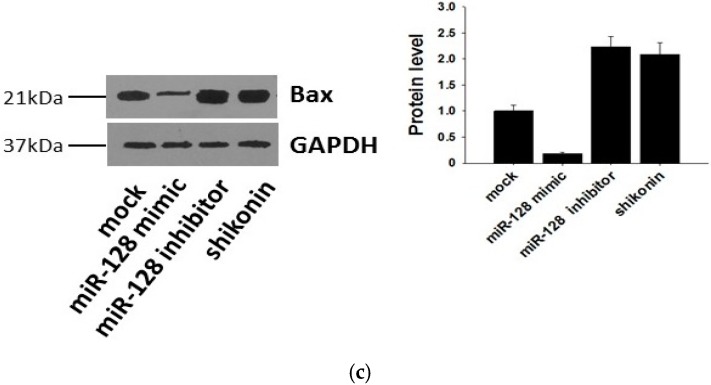
Bax was negatively regulated by exosomal miR-128 in MCF-7 recipient cells. (**a**) MiR-128 levels in secreted exosomes; (**b**) qRT-PCR detection of Bax mRNA in MCF-7 recipient cells after incubation with miR-128 knockdown/over-expressive or shikonin (1 µM treated for 24 h) treated exosomes; (**c**) Protein levels of Bax were detected by western blotting (left panel), and quantitative analysis of the western blotting result is shown in the right panel.

It has been reported that the target gene of miR-128 is Bax [28], which is a pivotal effector of the intrinsic or mitochondrial apoptosis pathway [29]. Knockdown of miR-128 significantly decreases the sensitivity of breast cancer cells to chemodrugs and promotes tumor proliferation. To verify the target gene of exosomal miR-128 in MCF-7 recipient cells, we transfected miR-128 mimics and inhibitors or stimulating cells with 1 µM shikonin to change the level of miR-128 in MCF-7 donor cell-derived exosomes. The expression of miR-128 in exosomes was measured by qRT-PCR (Figure 5a). MiR-128 knockdown/over-expressive or shikonin-treated exosomes were incubated with MCF-7 recipient cells for 24 h, and the mRNA as well as protein levels of Bax in recipient cells were detected by Western blotting and qRT-PCR. As shown in Figure 5b,c, exosomal miR-128 suppressed Bax expression in recipient MCF-7 cells.

## 3. Discussion

Previous studies on the shikonin functional mechanism have all focused on the intracellular pathway affected by shikonin. In the present study, we first demonstrate that shikonin can inhibit tumor proliferation by changing the tumor microenvironment via modulating exosome release in an extracellular pathway.

In recent years, with more research focused on cell secreted nanoparticles, exosomes have been regarded as a key factor in remodeling tumor-stromal interactions and promoting malignancy. Exosomes can efficiently deliver miRNAs into recipient cells, where they block the translation of their target genes and regulate recipient cell function [30]. For example, tumor-secreted miR-214 can be sufficiently delivered into recipient T cells, down-regulating phosphatase and tensin homologue (PTEN) and promoting Treg-mediated tumor immune evasion [31]. All of those studies focused on the function of exosomes moving from a donor cell to a different type of recipient cell. However, our study first demonstrates that exosomes derived from MCF-7 cells can be absorbed by this type of tumor cell itself and promote MCF-7 cell proliferation by the delivery of miR-128.

One interesting question is that because miR-128 facilitates MCF-7 cell proliferation by targeting the Bax gene, why can tumor cells selectively sort miR-128 into exosomes and release them? We propose an explanation that this is an insurance system of tumor cells. Each tumor cell releases part of its cellular miR-128 into exosomes to build a tumor microenvironment enriched with miR-128 containing exosomes. When a portion of the tumor cells absorbs some harmful substrate such as a chemodrug, they can quickly gain miR-128 from the microenvironment to downregulate the pro-apoptosis gene Bax, protecting themselves from programmed death. Another question is how MCF-7 cells selectively sort miR-128 into exosomes. A recent study reported that short sequence motifs over-represented in miRNAs can guide their loading into exosomes [32]. However, the mechanism behind the selective packaging of miRNAs into exosomes needs to be more clearly elucidated with further studies. 

## 4. Materials and Methods

### 4.1. Cells and Reagents

The human breast carcinoma MCF-7 cell line was purchased from the Institute of Biochemistry and Cell Biology, Shanghai Institutes for Biological Science, Chinese Academy of Sciences (Shanghai, China). Cells were maintained at 37 °C in a humidified 5% CO_2_ incubator in Dulbecco′s modified Eagle medium (DMEM) (Gibco, Waltham, MA, USA) that contained 10% fetal bovine serum, 100 units/mL of penicillin and 100 μg/mL of streptomycin. The miR-128 mimics and miRNA inhibitors were purchased from RIBOBIO (Guangzhou, China). Shikonin was purchased from Sigma-Aldrich (s7576). The anti-Tsg101 (ab133586) and anti-Bax (ab10813) antibodies for immunoblotting were purchased from Abcam (Hangzhou, China). The anti-CD9 (sc-9148) and anti-CD63 (sc-15363) antibodies were purchased from Santa Cruz (Shanghai, China).

### 4.2. Cell Proliferation Assay

Cell proliferation was measured with a CCK8 assay kit (Sigma-Aldrich, St. Louis, MO, USA). Briefly, MCF-7 cells were seeded into 96-well plates (Corning, Corning, NY, USA) at a density of 1 × 10^4^ cells per well in standard DMEM and incubated for 24 h under standard conditions (37 °C and 5% CO_2_). Then, the medium was replaced with either blank, serum-free DMEM or DMEM containing 0 mM, 0.01 µM, 0.1 µM, 1 µM, 10 µM or 100 µM shikonin for 24 h. The total volume in each well was 200 µL. MCF-7 cells were incubated in these solutions followed by treatment with 20 µL of CCK8 in each well for 1.5 h at 37 °C. Finally, the plates were shaken softly, and the optical density was recorded at 570 nm (OD 570) using an ELISA plate reader.

### 4.3. Isolation of Exosomes

Exosomes were collected from equivalent amounts of culture medium and conditioned by equivalent amounts of cells in triplicate cultures. When 80% confluency was reached, the cell layers were rinsed with DMEM and refreshed with DMEM containing 10% exosome-depleted FBS. The medium was harvested 24 h after cell culture or transfection, and the exosomes were isolated from the medium by the following three sequential centrifugation steps at 4 °C. First, centrifugation for 15 min at 500× *g* was performed to remove the cells, followed by 30 min at 10,000× *g* to remove the cell debris. The supernatant containing the cell-free culture media was transferred to a new tube and 0.5 volumes of the Total Exosome Isolation reagent (Invitrogen, Waltham, MA, USA, 4478359) was added. The culture media/reagent mixture was mixed well by vortexing, and the samples were incubated 4 °C overnight. After incubation, the samples were centrifuged at 10,000× *g* for 1 h at 4 °C. Exosomes were contained in the pellet at the bottom of the tube and then re-suspended in PBS for the following assays. 

### 4.4. Transmission Electron Microscopy Assay

For the TEM assay, the exosome samples were prepared as described above. Briefly, the exosome pellet was placed in a droplet of 2.5% glutaraldehyde in PBS buffer and fixed overnight at 4 °C. The exosome samples were rinsed 3 times in PBS for 10 min each and then fixed in 1% osmium tetroxide for 60 min at room temperature. Then, the samples were embedded in 10% gelatine, fixed in glutaraldehyde at 4 °C and cut into small blocks. The samples were dehydrated in increasing concentrations of alcohol. Then, the samples were placed in propylene oxide and infiltrated with increasing concentrations of Quetol-812 epoxy resin mixed with propylene oxide for 3 h per step. Finally, the samples were embedded in pure fresh Quetol-812 epoxy resin and polymerized at 35 °C for 12 h, 45 °C for 12 h and 60 °C for 24 h. Ultra-thin sections were cut using a Leica UC6 ultra-microtome and stained with uranyl acetate for 10 min followed by lead citrate for 5 min at room temperature. The samples were then observed with a transmission electron microscope (JEM-1010) at a voltage of 80 kV.

### 4.5. Nanoparticle Tracking Analysis (NTA)

The number and size of exosomes were directly tracked using the Nanosight NS 300 system (NanoSight technology, Malvern, UK) [25] configured with a 488 nm laser and a high-sensitivity sCMOS camera. Exosomes re-suspended in PBS at a concentration of 5 μg of protein/mL were further diluted 100- to 500-fold to achieve between 20–100 objects per frame. Samples were manually injected into the sample chamber at ambient temperature. Each sample was measured in triplicate at camera setting 13 with an acquisition time of 30 s and a detection threshold setting of 7. At least 200 completed tracks were analyzed per video. The NTA analytical software version 2.3 was used for capturing and analyzing the data.

### 4.6. Immunofluorescence

Cells were cultured on 4-well chamber slides. At the time of harvest, cells were fixed with 4% paraformaldehyde and then permeabilized with 0.01% Triton X-100 for 10 min. All samples were treated with DAPI dye for nuclear staining (358 nm). For confocal microscopy, a Nikon C2 Plus confocal microscope was used.

### 4.7. Transfection of Cells with miRNA Inhibitor and Mimic

Cells were seeded in 6-well plates or 10-mm dishes and transfected the following day using Lipofectamine 2000 (Invitrogen) according to the manufacturer’s instructions. For the transfection, 20 pmol RNA per 10^5^ cells was used. Cells were harvested 48 h after transfection for real-time PCR analysis and Western blotting.

### 4.8. RNA Isolation and qRT-PCR of mRNA and Mature miRNAs

Total cellular RNA was extracted using a miRNeasy Mini Kit (QIAGEN, Shanghai, China). The qRT-PCR was performed using TaqMan probes (Applied Biosystems, Grand Island, NY, USA) for mature miRNAs or SYBR Green (Takara, Mountain View, CA, USA) for mRNA. Briefly, total RNA was reverse-transcribed to cDNA using AMV reverse transcriptase (Takara) and a stem-loop RT primer or reverse primer (Applied Biosystems). Real-time PCR was performed on an Applied Biosystems 7900 Sequence Detection System (Applied Biosystems). All of the reactions, including the no-template controls, were run in triplicate. After the reactions, the CT values were determined using fixed threshold settings. The miRNA expression in the cells was normalized to U6 snRNA, and mRNA expression in the cells was normalized to GAPDH.

### 4.9. Immunoblotting

Cells were lysed with lysis buffer (20 mM Tris-HCl, 150 mM NaCl, 0.5% Nonidet P-40, 2 mM EDTA, 0.5 mM DTT, 1 mM NaF, 1 mM PMSF and 1% Protease Inhibitor Cocktail from Sigma, pH = 7.5) for 30 min on ice. The lysates were cleared by centrifugation (16,000× *g*) for 10 min at 4 °C and then used for the immunoblotting assay. Bax protein levels were quantified by Western blotting analysis using antibodies against Bax (abcam, ab10813). Normalization was conducted by blotting the same samples with an antibody against GAPDH (Santa Cruz, sc-365062, Shanghai, China).

### 4.10. Statistical Analysis

All of the images of the Western blotting and qRT-PCR assays were representative of at least three independent experiments. The qRT-PCR was performed in triplicate. The data are presented as the mean ± SD for three or more independent experiments. The differences were considered to be statistically significant at *p* < 0.05 assessed using Student’s *t-*test.

## 5. Conclusions

The present work provides evidence that shikonin inhibits the proliferation of MCF-7 cells through reducing tumor-derived exosomes. Exosomes secreted by donor MCF-7 cells containing miR-128 can be absorbed by MCF-7 recipient cells. Exosomal miR-128 can downregulate the Bax gene in recipient MCF-7 cells and promote cell proliferation. Thus, decreased exosome secretion by shikonin treatment can suppress MCF-7 cell proliferation.
